# Hyperbaric oxygen therapy alleviates vascular dysfunction and amyloid burden in an Alzheimer’s disease mouse model and in elderly patients

**DOI:** 10.18632/aging.203485

**Published:** 2021-09-09

**Authors:** Ronit Shapira, Amos Gdalyahu, Irit Gottfried, Efrat Sasson, Amir Hadanny, Shai Efrati, Pablo Blinder, Uri Ashery

**Affiliations:** 1 School of Neurobiology, Biochemistry and Biophysics, The George S. Wise Faculty of Life Sciences, Tel Aviv University, Tel-Aviv, Israel; 2 Sagol School of Neuroscience, Tel Aviv University, Tel-Aviv, Israel; 3 Sackler School of Medicine, Tel Aviv University, Tel-Aviv, Israel; 4 Sagol Center for Hyperbaric Medicine and Research, Assaf Harofeh Medical Center, Be’er Ya’akov, Israel

**Keywords:** Alzheimer's disease, hyperbaric oxygen therapy, vascular dysfunction, cerebral blood flow, amyloid burden

## Abstract

Vascular dysfunction is entwined with aging and in the pathogenesis of Alzheimer’s disease (AD) and contributes to reduced cerebral blood flow (CBF) and consequently, hypoxia. Hyperbaric oxygen therapy (HBOT) is in clinical use for a wide range of medical conditions. In the current study, we exposed 5XFAD mice, a well-studied AD model that presents impaired cognitive abilities, to HBOT and then investigated the therapeutical effects using two-photon live animal imaging, behavioral tasks, and biochemical and histological analysis. HBOT increased arteriolar luminal diameter and elevated CBF, thus contributing to reduced hypoxia. Furthermore, HBOT reduced amyloid burden by reducing the volume of pre-existing plaques and attenuating the formation of new ones. This was associated with changes in amyloid precursor protein processing, elevated degradation and clearance of Aß protein and improved behavior of 5XFAD mice. Hence, our findings are consistent with the effects of HBOT being mediated partially through a persistent structural change in blood vessels that reduces brain hypoxia. Motivated by these findings, we exposed elderly patients with significant memory loss at baseline to HBOT and observed an increase in CBF and improvement in cognitive performances. This study demonstrates HBOT efficacy in hypoxia-related neurological conditions, particularly in AD and aging.

## INTRODUCTION

In recent years, it has become clear that vascular dysfunction is entwined in the pathogenesis of Alzheimer’s disease (AD) and cognitive decline during aging [1–3]. Vascular risk factors, such as obesity, diabetes, atherosclerosis, smoking and hypertension, are major risk factors for AD [4]. Cerebral amyloid angiopathy (CAA), the deposition of Aβ peptide in cerebral vessel walls, is the most common vascular pathology in AD [5, 6]. Both AD and CAA are associated with reduced cerebral blood flow (CBF), which precedes the clinical onset of dementia [7–10] and correlates with the degree of cognitive impairment in AD [1, 11]. Accordingly, CBF has been proposed as a marker for disease severity [12]. Cerebral hypoperfusion [13–15] and the cerebral hypoxia that ensues [16, 17] have also been detected in animal models of AD. This hypoperfusion has been attributed to several mechanisms, including reduced vascular density [18, 19], constriction of cerebral arterioles [20, 21] and impaired neurovascular coupling [22, 23]. Cerebral hypoperfusion is also associated with accelerated cognitive decline [3, 24] and increased risk of dementia in the general population [2]. Therefore, developing treatments that target vascular dysfunction, as well as other AD pathologies, could be a promising avenue for treating the disease and improving cognitive performances in healthy elderly populations suffering from cognitive decline.

Hyperbaric oxygen therapy (HBOT), the medical administration of 100% oxygen at environmental pressure greater than one atmosphere absolute (ATA) [25], is in clinical use for a wide range of medical conditions. At present, there are only 13 FDA-approved indications for HBOT, including non-healing ischemic wounds, post radiation injuries, decompression sickness, burn repair, carbon monoxide intoxication, and diabetic ulcers [26]. In addition, there is a growing number of off-label treatments [27] like usages of HBOT to induce neuroplasticity and improve neuro-cognitive functions in post-traumatic brain injuries (TBI) or post-stroke patients [28, 29]. Further clinical trials that are being performed these days and additional basic scientific studies aiming to understand HBOT’s mechanisms of action, will most probably expand the use of HBOT to other areas.

By increasing the dissolved oxygen content of the blood, HBOT can sustain tissues with minimal perfusion [25, 30]. Evidence from clinical studies demonstrated that HBOT induces recovery of cognitive functions in post-TBI patients [31, 32] by inducing cerebral angiogenesis, increasing cerebral blood flow and volume, and improving cerebral white and grey microstructures [33]. Elevation of CBF and restoration of physical abilities and cognitive functions were also shown in stroke patients [34, 35]. However, our ability to investigate the underlying mechanisms of these HBOT-mediated effects in patients is very limited. At the same time, animal models offer major advantages in advancing our understanding of the cellular and molecular mechanisms leading to increased CBF.

Recently, it was shown that HBOT improved cognitive performance in animal models of Alzheimer’s disease [17, 36], and improved the metabolic status and cognitive scores of AD and amnestic mild cognitive impairment patients [37–39]. However, it is not known if HBOT mitigates cerebrovascular dysfunction in AD. Therefore, we investigated the effects of HBOT on CBF and cognitive decline in the 5XFAD mouse model of AD that presents aggressive accumulation of amyloid load [40], cerebrovascular abnormalities [15, 41, 42] and cognitive impairment [40], as well as in elderly individuals suffering from significant memory loss. We report that HBOT improves CBF and cognitive function in both AD mice and elderly patients with significant memory loss. HBOT elevated CBF and reduced cerebral hypoxia by increasing blood vessel diameter. Furthermore, by tracking single plaques *in vivo* over weeks, we show for the first time that HBOT reduces the volume of pre-existing plaques and the appearance of newly-formed plaques.

## RESULTS

### HBOT reduces the amyloid load of 5XFAD mice by reducing the number of newly-formed plaques and decreasing the volume of existing plaques

**Figure 1:**
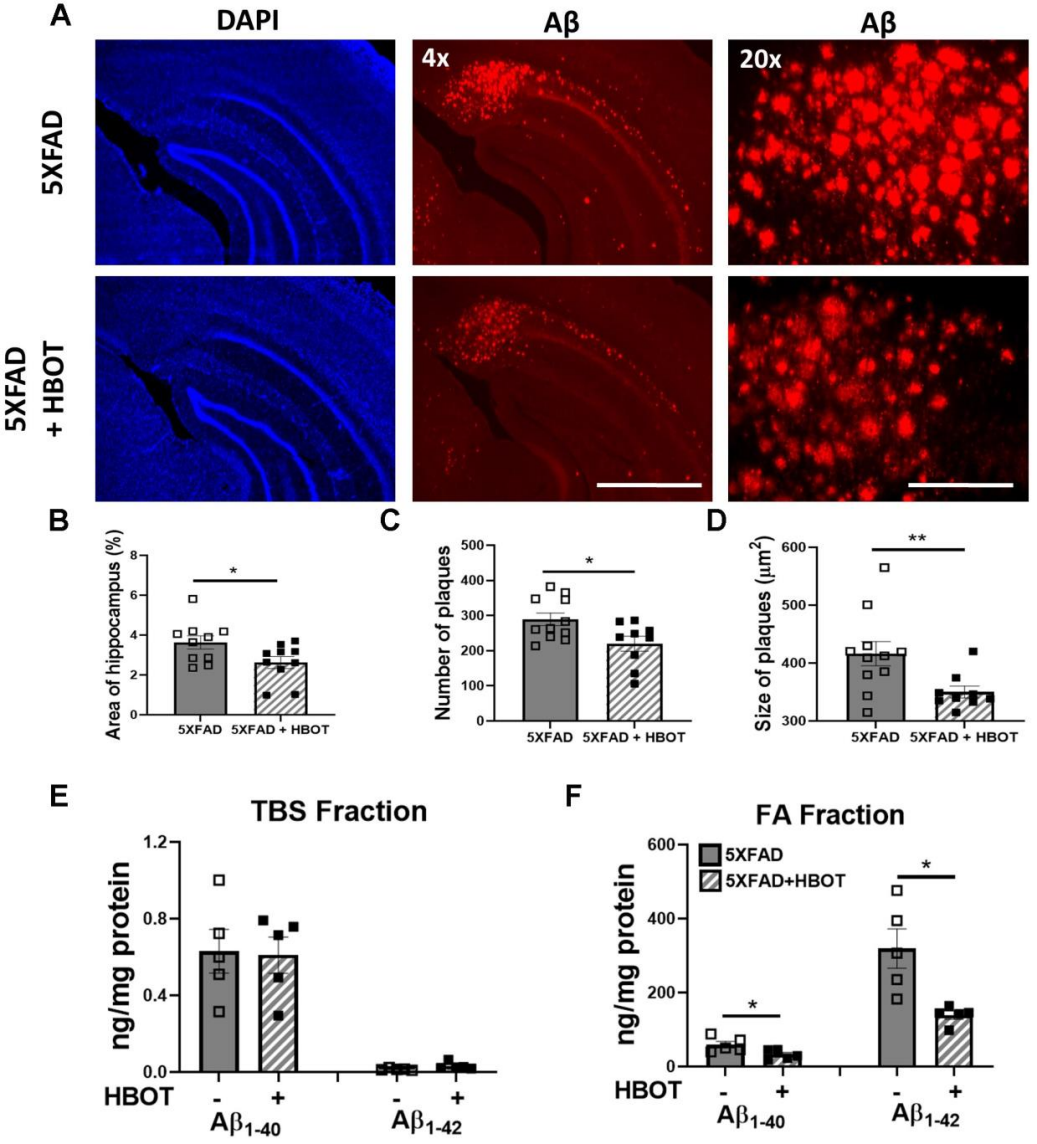
**HBOT reduces amyloid plaques in the hippocampal area of 6-month old 5XFAD mice.** Amyloid plaques were visualized by immunostaining with anti-Aβ antibodies (4G8). (**A**) Representative images of Aβ in the hippocampal field of HBO-treated 5XFAD (n=10, lower panel) and control 5XFAD mice (n=10, upper panel); left and middle panels, x4 magnification, scale bar: 1000 μm; right panel, x20 magnification, scale bar: 200 μm. (**B**) Quantification of the percentage of hippocampal area occupied by plaques. (**C**) Number of plaques. (**D**) Mean size of plaques. (**E**, **F**) Soluble Aβ was initially extracted from hippocampi with TBS by ultracentrifugation and then insoluble Aβ was extracted with 70% formic acid (FA) after ultra-centrifugation. ELISA analysis of soluble (**E**) and insoluble (**F**) Aβ40 and Aβ42 in hippocampal lysates of HBO-treated 5XFAD and control 5XFAD mice (n = 5/group). (**B**, **C**, **F**) -t-test, (**D**, **F**)- welch correction t-test. Values represent means ± SEM. *P < 0.05, ** *P* < 0.01.

We first asked if increasing oxygen delivery to the brain by administrating HBOT reduced amyloid burden. To that end, we employed a custom-made HBO chamber to expose 6 month-old 5XFAD and wild type (wt) mice to HBOT at 2 ATA for 60 minutes per day, 5 days a week for 4 weeks (i.e., 20 treatments). To assess the effect of HBOT on amyloid burden in the treated 5XFAD mice, brains were stained with anti-Aβ pan-antibodies (4G8, directed against epitope 17–24; Figure 1). We found significant reduction in amyloid burden in the hippocampus of HBO-treated 5XFAD mice, as manifested by the reduced percentage of hippocampal area displaying 4G8 immunoreactivity (-54.32%, *P*=0.0353; Figure 1A, 1B), decreased numbers of plaques (-31.58%, *P*=0.0217; Figure 1C) and smaller plaque size (-18.94%, *P* = 0.0125 by Welch's correction; Figure 1D), relative to control 5XFAD mice exposed to normobaric conditions.

We then analyzed the levels of soluble (TBS fraction) and insoluble (Formic Acid fraction) levels of Aβ42 and Aβ40 in the hippocampus by ELISA (Figure 1E, 1F). Following 1 month of HBOT, levels of insoluble Aβ42 were reduced by ~56% (FA fraction, *P* = 0.0292 by Welch's correction; Figure 1F), while Aβ40 levels were reduced by ~45% (FA fraction, *P* = 0.0356; Figure 1F) in HBO-treated 5XFAD mice compared with control 5XFAD mice. In contrast, soluble Aβ42 and Aβ40 levels were unchanged (TBS fraction; Figure 1E). Collectively, these data demonstrate that HBOT reduced the amyloid load in the hippocampal formation of 5XFAD mice.

**Figure 2:**
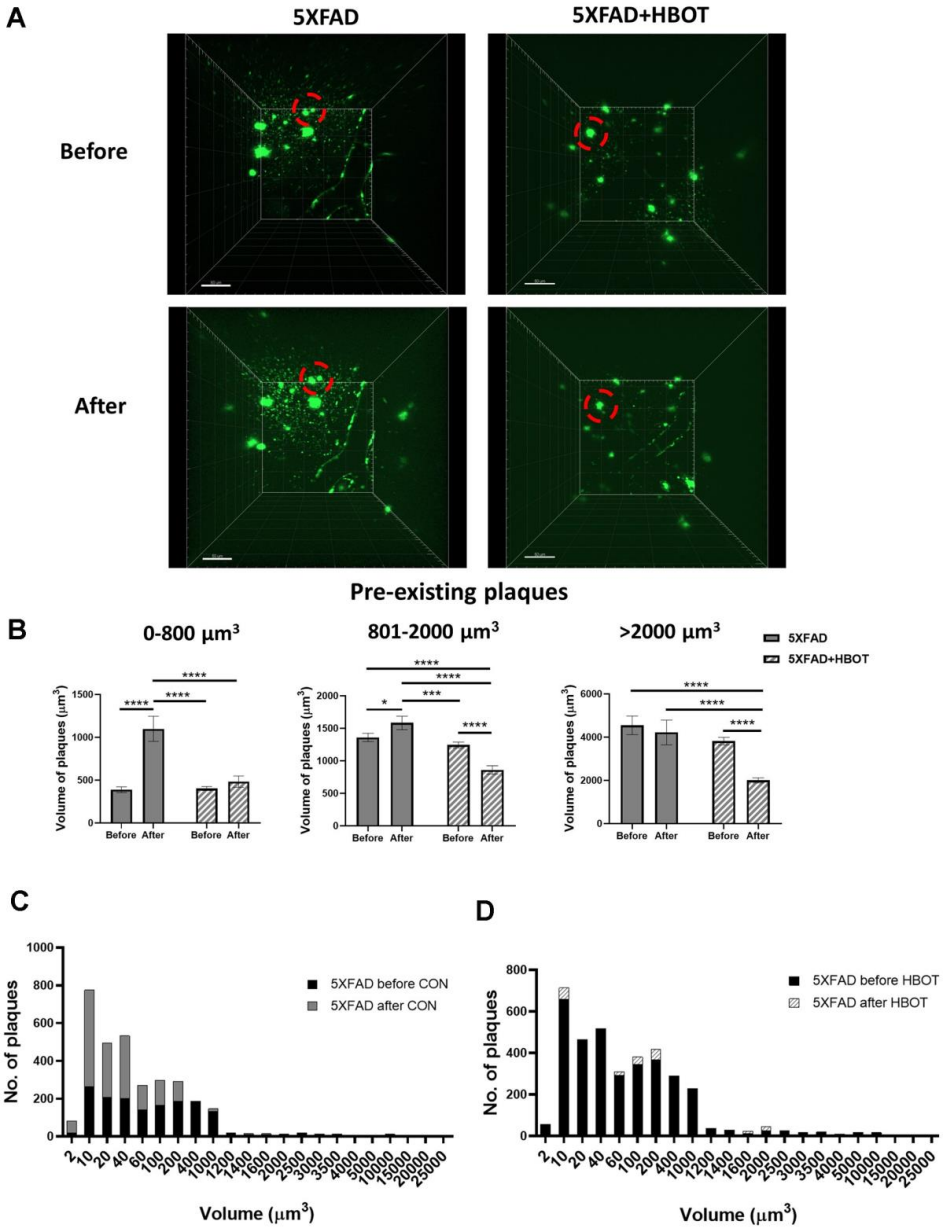
**HBOT reduces the population of newly-formed plaques and reduces the volume of pre-existing plaques.** Amyloid plaques were visualized *in vivo* using two-photon microscopy imaging in live animals by injecting methoxy-X04 24 h before every imaging session. (**A**) Representative images of plaques in the somatosensory cortex of HBO-treated 5XFAD (n=4, right panel) and control 5XFAD mice (n=3, left panel) before (upper panel) and after 1 month of treatment (lower panel); red circles indicate the change in specific plaques, scale bar: 50 μm. (**B**) Analysis of the volume of pre-existing plaques before and after each treatment in the same animal, categorized according to initial plaque size. (**C**, **D**) Distribution of plaque populations by volume in control 5XFAD (**C**; before: N=1619, after: N=3180) and HBO-treated 5XFAD mice (**D**; before: N=3425, after: N=3524). Two-way ANOVA with repeated measures and post-hoc Fisher LSD tests were performed. Values represent means ± SEM. *P < 0.05, *** *P* < 0.001, **** *P* < 0.0001.

To study the dynamics of plaque formation and growth *in vivo*, we addressed changes in amyloid plaques before and after HBOT of the same mice by performing longitudinal *in vivo* two-photon imaging via introduction of a cranial window over the barrel cortex [43]. We then stained amyloid plaques *in vivo* with methoxy-X04 and imaged the same Aβ plaques before and after one month of exposure to HBOT or control conditions. Initially, we investigated whether HBOT affected the volume of pre-existing plaques by tracing the volume of single plaques before and after each treatment in the same animals (Figure 2A, 2B).

We classified pre-existing plaques into sub-populations according to their initial volume (Figure 2B) and evaluated the effect of HBOT on these groups. We found that without treatment, small pre-existing plaques ranging in volume from 2-800 μm^3^ exhibited a greater increment in volume (2.83-fold) than did larger pre-existing plaques. Larger plaques, with volumes ranging 801-2000 μm^3^ (medium-sized plaques) showed only a 1.17-fold increase and plaques with sized >2001 μm^3^ (large plaques) showed no increase in volume (Figure 2B). This is in accordance with previous studies that demonstrated that smaller plaques present a higher rate of increased volume, relative to larger plaques in AD models [44, 45]. Strikingly, HBOT halted the significant increase in volume of the small pre-existing plaques (*P* = 0.3387; Figure 2B, left panel) and facilitated reductions in the volumes of medium-sized (*P* < 0.0001; Figure 2B, middle panel) and large plaques (*P* < 0.0000001, Figure 2B, right panel). It should be noted that the initial averaged volume of pre-existing plaques was similar between treatment groups at all volume ranges (Figure 2B), yet HBOT had differential effects on the growth of smaller plaques and the breakdown of larger plaques. Averaging all plaque sizes uncovered that in control 5XFAD mice, existing plaques increased in size by 12.3% on average over 1 month (*P =* 0.0499), while existing plaques in HBO-treated 5XFAD mice decreased by 40.05% on average (*P <* 0.001, two-way ANOVA with repeated measures) (data not shown).

We next analyzed the total number of plaques to learn about pre-existing and novel plaques and found that in 5XFAD mice the number of plaques nearly doubled over the course of one month (1.96-fold change), suggesting that high and rapid synthesis of newly-formed plaques had occurred (Figure 2C). However, in HBO-treated 5XFAD mice, the number of plaques was unchanged over the same period (1.03-fold change; Figure 2D). Assessing the distribution of plaque volumes revealed that while there was a dramatic increase in that plaque population with volumes ranging from 2-400 μm^3^ in control 5XFAD mice over the course of a month, in HBO-treated 5XFAD mice, the distribution of plaque volumes did not change during a month of treatment (Figure 2C, 2D).

Taken together, these results suggest that HBOT both attenuates the appearance of newly- formed plaques and causes a reduction in the volume of pre-existing plaques.

### HBOT reduces abnormal processing of amyloid precursor protein and increases levels of Aβ degradation and clearance

**Figure 3:**
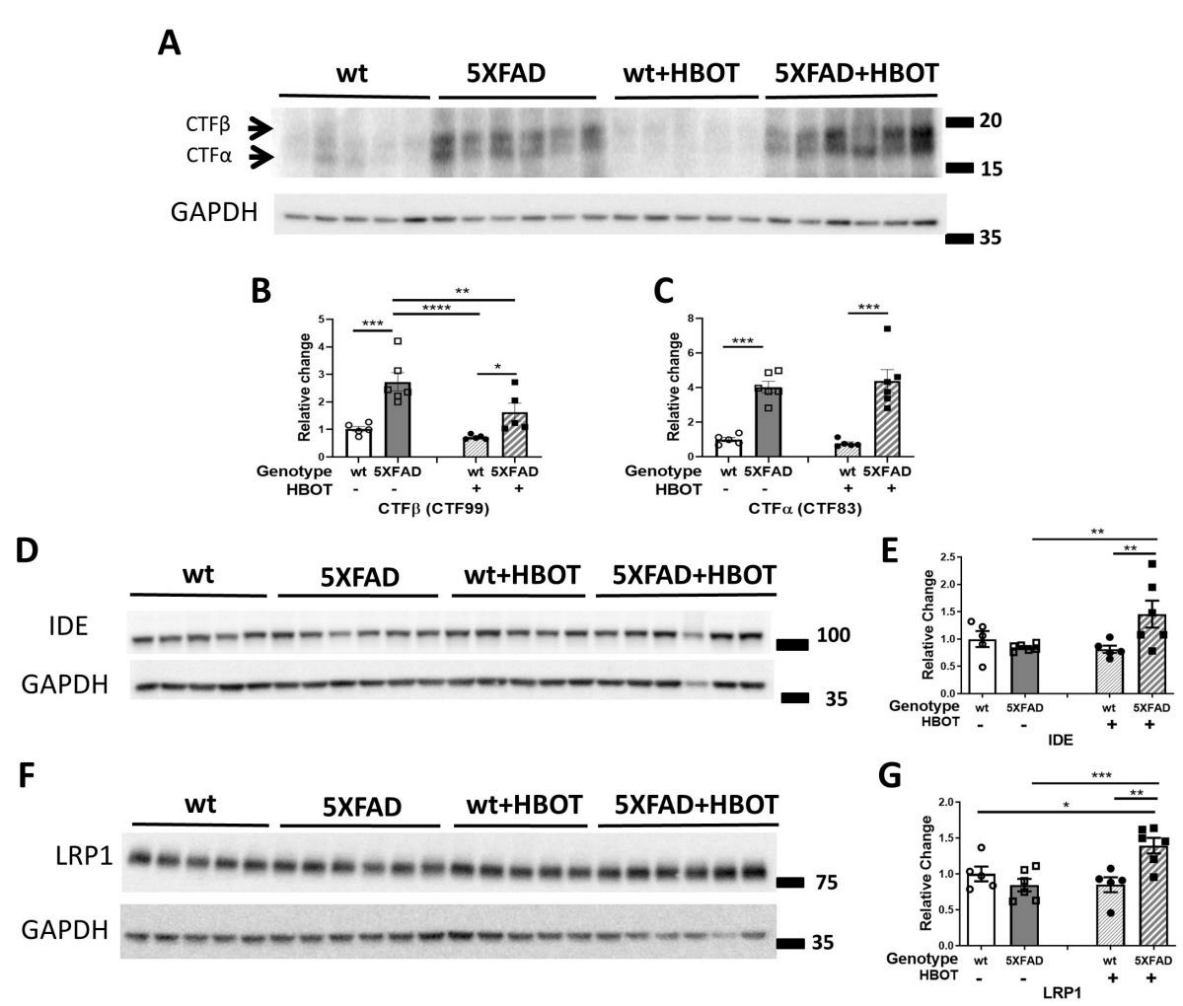
**HBOT reduces abnormal processing of APP and attenuates Aβ degradation and clearance in 5XFAD mice.** (**A**) Representative immunoblot assays of the carboxyl-terminal fragment (CTF)β and CTFα. (**B**, **C**) Quantification of western blots in (**A**), presented as percentages of wt control, normalized to GAPDH levels (n = 5–6/group). (**D**–**G**) Representative immunoblot assays of IDE protein (**D**) and LRP1 in (**F**). (**E**, **G**) Quantification of western blots in (**D**, **F**), respectively, presented as percentages of wt controls, normalized to GAPDH levels (n = 5-6/group). Two-way ANOVA and post-hoc Fisher LSD tests were performed. Values represent means ± SEM. * *P* < 0.05, ** *P*<0.01, *** *P* < 0.001, **** *P* < 0.0001.

To understand the molecular mechanisms that contribute to the observed reduction in amyloid burden, we next assessed key proteins involved in amyloid precursor protein (APP) processing, and Aβ degradation and clearance. First, levels of the β-secretase-cleaved C-terminal fragment of APP (β-CTF or C99) and the α-secretase-cleaved C-terminal fragment of APP (α- CTF or C83) were measured [46]. HBO treatment significantly reduced C99 levels in 5XFAD mice (-40.41%, *P =* 0.0060; Figure 3A, 3B) and induced no change in levels of the C83 fragment in 5XFAD mice (*P =* 0.7198; Figure 3A, 3C), suggesting that HBOT reduced β-secretase (BACE1)-mediated cleavage of APP. Indeed, BACE1 levels were found to be reduced in the HBO-treated mice (Supplementary Figure 1C, 1D). As we previously showed with the 3xTg mouse model [17], the levels of full-length APP were also unchanged upon HBO treatment of 5XFAD mice (Supplementary Figure 1A, 1B), whereas the levels of the α-secretase ADAM10 were reduced by HBOT (Supplementary Figure 1C–1E). In addition, while the levels of presenilin 1 (PSE), a component of the γ-secretase complex, were also significantly reduced in 5XFAD mice following HBOT (Supplementary Figure 1C–1E), no changes were found in the levels of nicastrin, another component of the γ-secretase complex (Supplementary Figure 1C, 1E).

HBOT has been shown to affect microglial function and increase Aβ clearance, thus contributing to neuroprotection [17, 47]. Analysis of plaque-associated microglia showed that following HBOT, the number of microglia per plaque increased, suggesting that HBOT induced microglial recruitment to the plaques, possibly supporting plaque degradation (Supplementary Figure 1F).

We next examined whether HBO treatment affects insulin-degrading enzyme (IDE), a key enzyme responsible for the degradation of Aβ peptides [48]. IDE levels were significantly increased in HBO-treated 5XFAD mice, as compared to controls (+72.58%, *P =* 0.009; Figure 3D, 3E), suggesting that HBOT increased Aβ degradation. Low density lipoprotein receptor-related protein 1 (LRP1) plays a role in clearing Aβ from the brain, across the blood-brain barrier and into the systemic circulation [49, 50] or clearing Aβ from the parenchyma into neurons [51] and astrocytes [52]. LRP1 levels were significantly increased in HBO-treated 5XFAD mice, as compared to controls (+65.16%, *P =* 0.0008; Figure 3F, 3G), suggesting that HBOT increases Aβ clearance.

Taken together, these results suggest that HBOT attenuates amyloid burden by reducing Aβ synthesis via a decrease in APP-cleaving enzymes and by enhancing Aβ elimination via increased activities of degradation and clearance pathways.

### HBOT alleviates the reduction in vessel diameter and increases blood flow and arteriolar lumen size in 5XFAD mice

**Figure 4:**
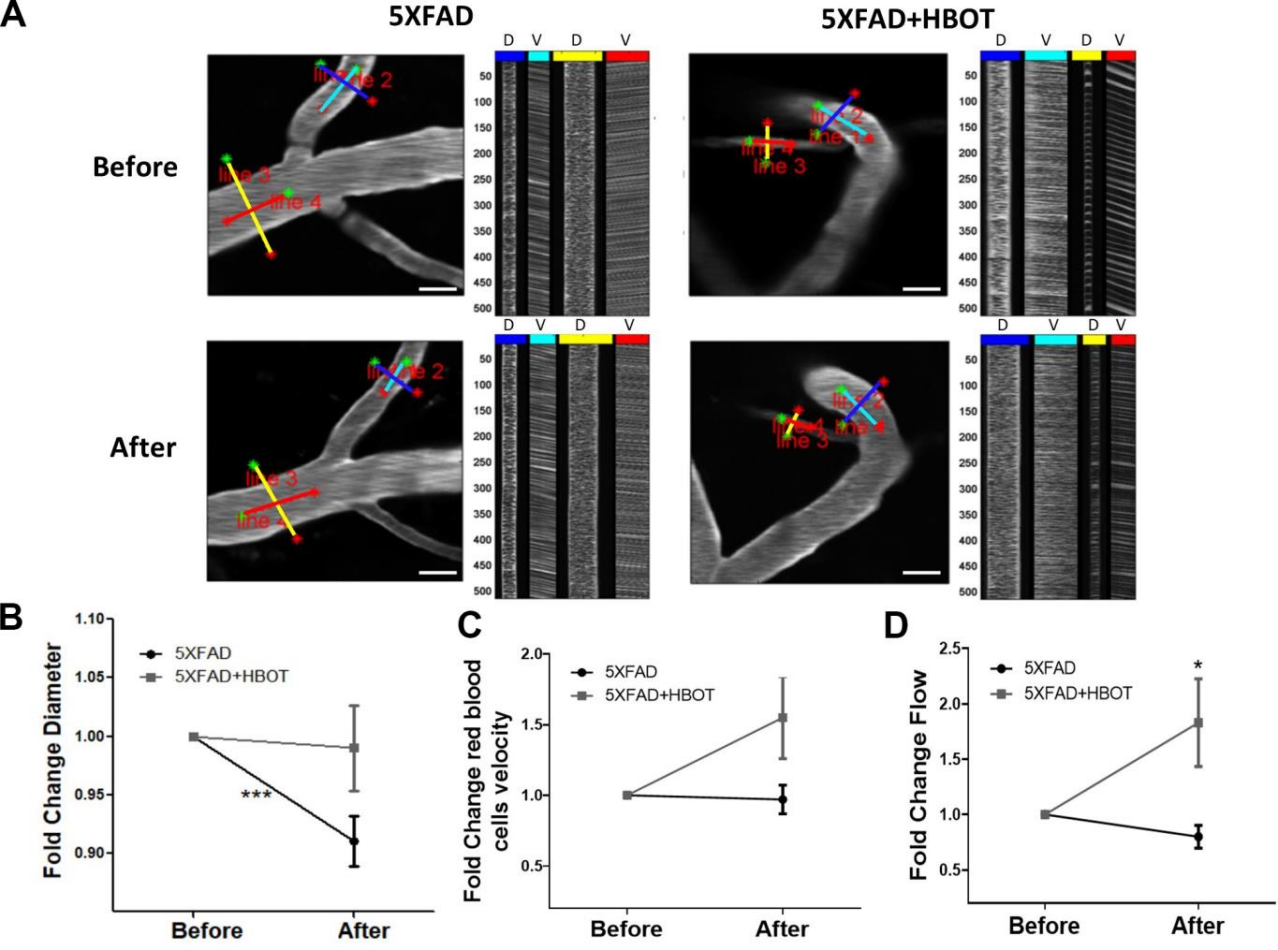
**HBOT alleviates the reduction in vessel diameter in 5XFAD mice and increases blood flux.***In vivo* two-photon microscopy imaging and measurements of diameter and velocity in blood vessels of the somatosensory cortex in 5XFAD mice using spatially optimized line scans. (**A**) Representative images of fluorescently stained vessels of the somatosensory cortex of an HBO-treated 5XFAD mouse (right panel) and a control 5XFAD mouse (left panel) before (upper panel) and after a month of treatment (lower panel). Line scan patterns are superimposed on the vessels. Lines of the scan path along the length were used to calculate RBC velocity (V), while lines across the diameter of the vessels were used to calculate diameter (**D**). The line scans generated from the path can be stacked sequentially as a function of time to produce a raw cascade image (right of each image). Vessel diameter was calculated as the full width at half-maximum of a time average of several scans across the width of a vessel. RBC velocity was calculated from the angle of the RBC streaks. (**B**–**D**) Quantification of vessel diameter (**B**), RBC velocity in the blood vessels (**C**) and RBC flow (**D**), normalized to each treatment group baseline value. Paired t-tests and student t-tests were performed. Values represent means ± SEM.* *P* < 0.05, *** *P* < 0.001.

Abnormalities in microvessels found near Aβ plaque-deposited areas in 5XFAD mice contribute to reduced CBF [15]. To gain insight into the effects of HBOT on CBF, we used *in vivo* two-photon microscopy to address the cortical vasculature of the same mouse before and after a month of exposure to HBOT or control conditions (Figure 4). Such analysis revealed that in control 5XFAD mice, there was significant reduction in vessel diameters over the course of the month (-8.57%, *P* = 0.0002, paired t-test; Figure 4A, 4B). In contrast, no significant reduction in blood vessel diameters was observed in HBO-treated 5XFAD mice (-0.53%, Figure 4A, 4B, *P* = 0.2206, paired t-test). The fold change in vessel diameters showed a downward trend in the control group, as compared to the HBO-treated group (*P* = 0.0601, t-test; Figure 4B).

To measure blood flow velocity in specific blood vessels, the vascular serum was labeled with a fluorescent dye (FITC) conjugated to high molecular weight dextran to prevent dye leakage from the vasculature. The velocities of non-fluorescent red blood cells (RBCs) were then tracked on this fluorescent background by tracing RBC movement over distance and time [53, 54]. RBC velocity was elevated following one month of HBOT, as compared to control normobaric conditions, yet not significantly (control: -2.70%; HBOT: +54.89%; fold change *P* = 0.0652, t-test; Figure 4C). However, RBC flow, which provides a complete description of blood flow in each vessel, showed significant improvement following HBOT (control: -20.35%, HBOT: +82.82%; fold change *P* = 0.014, t-test; Figure 4D). These results directly show that HBOT alleviated reductions in blood vessel diameter, and, therefore, contributed to increased blood flow in 5XFAD mice.

**Figure 5:**
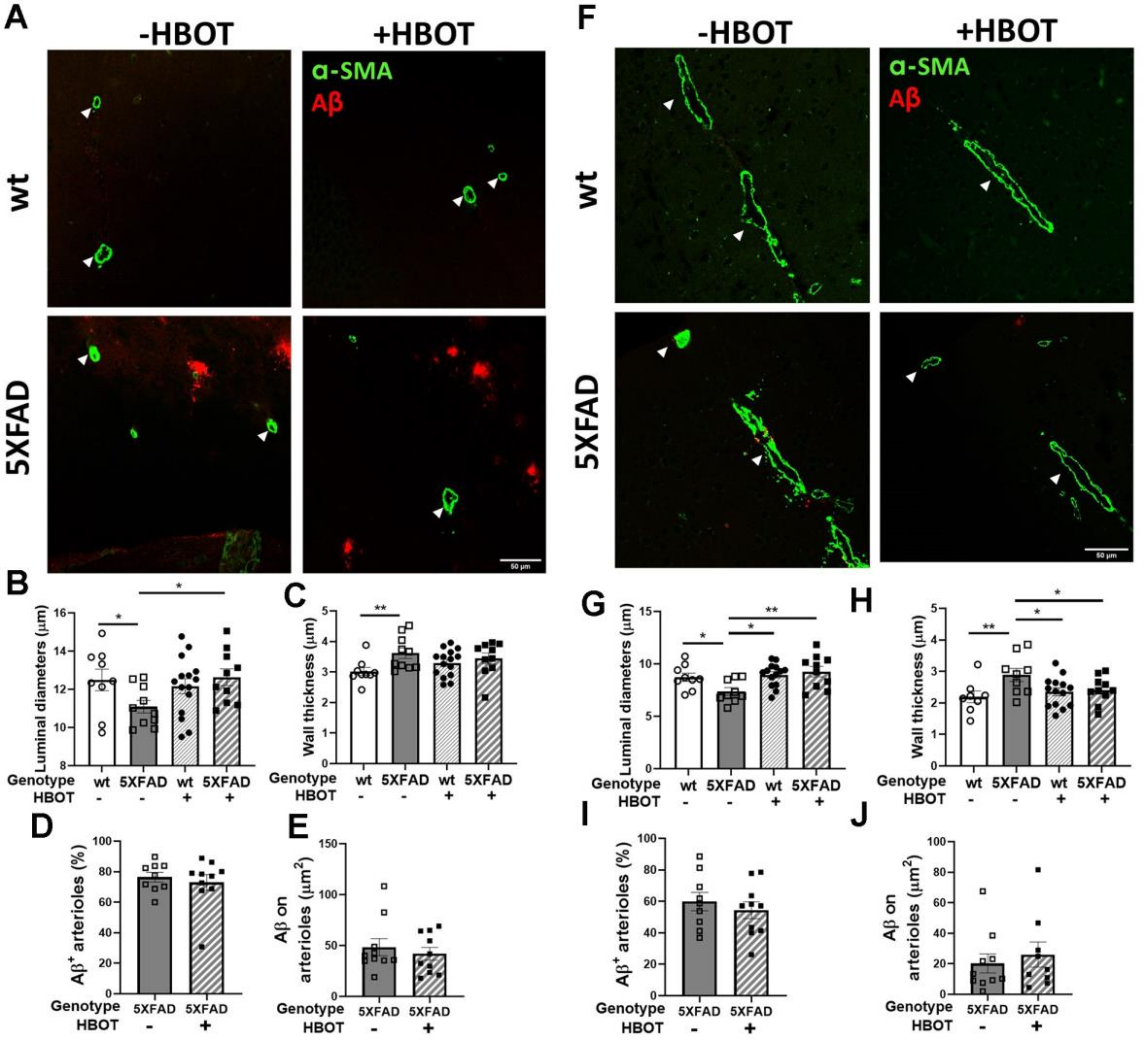
**HBOT attenuates arteriolar luminal diameter but not amyloid deposition around arterioles in 6-month old 5XFAD mice.** Arterioles were visualized using immunostaining with anti-SMA antibody while vascular amyloid deposition was visualized using anti-Aβ antibody (4G8). (**A**, **F**) Representative images of arterioles and Aβ in hippocampal (**A**) and cortical fields (**F**) of HBO-treated wt (n=9, upper right panels) and 5XFAD mice (n=10, lower right panels) and control wt (n=9, upper left panels) and 5XFAD mice (n=10, lower left panels) (x40 magnification, scale bar: 50 μm). White arrows show hippocampal and cortical arterioles. (**B**–**E**) and (**G**–**J**), Quantification of arteriolar luminal diameters (**B**, **G**), arteriolar wall thickness (**C**, **H**) and percentages of arterioles that stained positive for Aβ (**D**, **I**) and Aβ deposition area around arterioles (**E**, **J**) in the hippocampal (**B**–**E**) and cortical fields (**G**–**J**). Two-way ANOVA and post-hoc Fisher LSD tests were performed. Values represent means ± SEM. * *P* < 0.05, ** *P* < 0.01.

Finally, we double-stained the vasculature using antibodies raised against anti-smooth muscle actin antibody (α-SMA antibody) and Aβ (4G8 antibody) and measured arteriolar wall thickness, luminal diameter and the Aβ area around arterioles in hippocampal and cortical areas in the brains of mice exposed to HBOT or normobaric conditions (Figure 5). In both the hippocampal and cortical areas, SMA staining revealed that control 5XFAD mice displayed decreased luminal diameters (hippocampus: -11.22%, *P* = 0.0107; cortex: -17.14%, *P* = 0.0407; Figure 5A, 5B, 5F, 5G, respectively), higher arteriolar wall thickness (hippocampus: +19.75%, *P* = 0.0308; cortex: +31.74%, *P* = 0.0077; Figure 5A, 5C, 5F, 5H, respectively), and high Aβ deposition around arterioles, as compared to wt mice. Similar changes were observed in AD patients, as compared to non-AD controls [55, 56]. Arteriolar luminal diameter reduction in particular has been suggested to play a role in cerebral hypoperfusion during AD progression [56]. Following HBOT, the luminal diameter was greatly increased in both hippocampal (+13.798%, *P* = 0.016; Figure 5A, 5B) and cortical areas (+25.59%, *P* = 0.0064; Figure 5F, 5G) of HBO-treated 5XFAD mice, as compared to control mice. HBO treatment did not change arteriolar wall thickness of 5XFAD mice in hippocampal areas (*P* = 0.4387; Figure 5A, 5C), yet caused a decrease in wall thickness in cortical areas of treated 5XFAD mice, as compared to control mice (-16.56%, *P* = 0.0477; Figure 5F, 5G). Finally, no changes were measured in Aβ deposition around arterioles in both hippocampal (Figure 5D, 5E) and cortical areas (Figure 5I, 5J) following HBOT. These findings corroborate our *in vivo* imaging results showing a narrowing of blood vessels in 5XFAD mice over a month (Figure 4) and suggest a mitigating effect of HBOT on vascular parameters. Collectively, these results suggest improved blood perfusion following HBOT.

### HBOT reduces hypoxia and hypoxia inducible factor-1 (HIF-1) levels in 5XFAD mice

**Figure 6:**
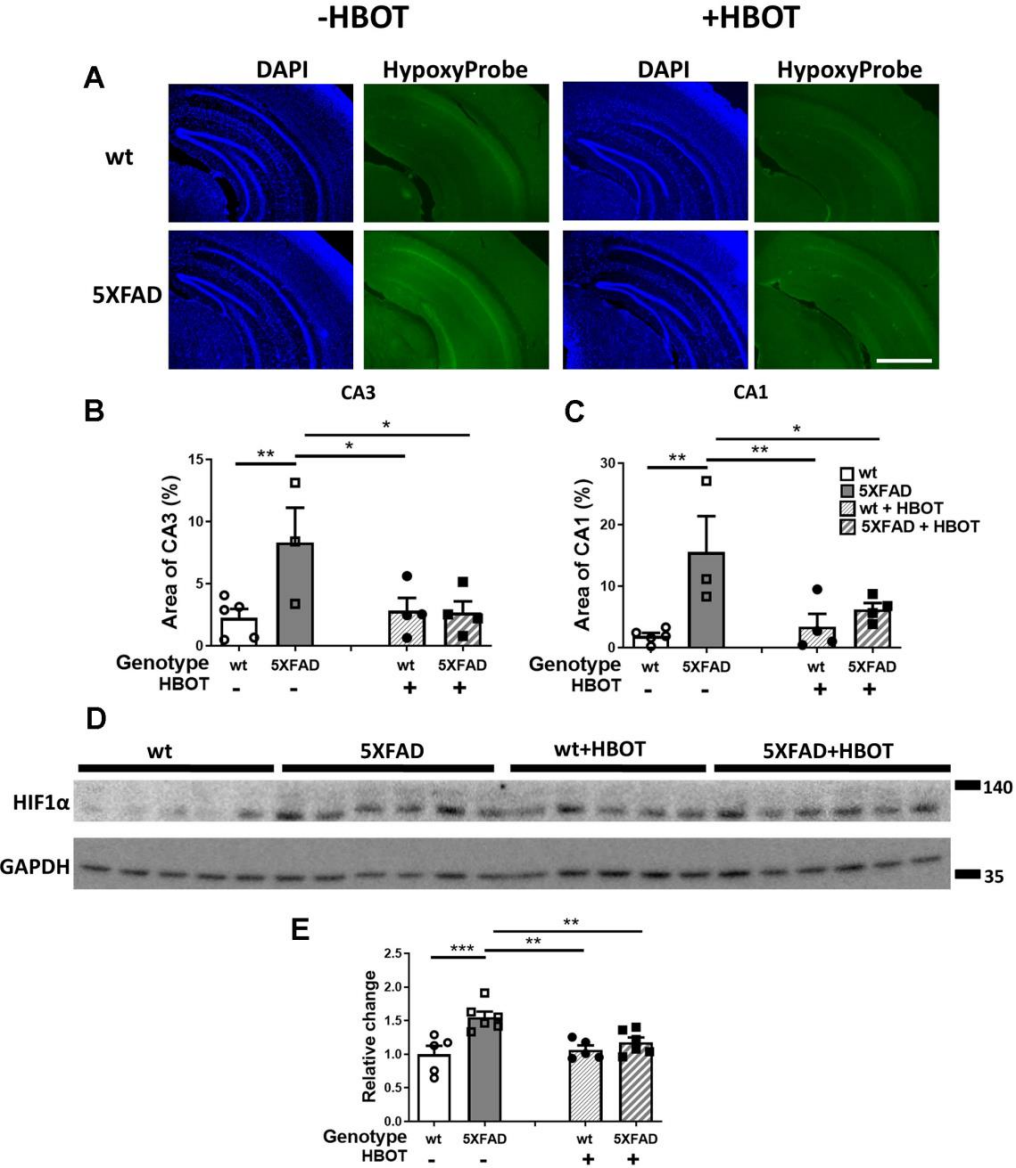
**HBOT reduces hypoxia and HIF1α transcription factor levels in the hippocampal area of 6-month old 5XFAD mice.** (**A**) Uptake of Hypoxyprobe by low oxygen-bearing cells was visualized by immunostaining. Representative images of the presence of hypoxia in the hippocampal field of HBO-treated wt (n=4, right upper panel) and 5XFAD mice (n=4, right lower panel), and control wt (n=5, left upper panel) and 5XFAD mice (n=3, left lower panel); x4 magnification, scale bar: 1000 μm. (**B**, **C**) Quantification of the percentage of the CA3 (**B**) and CA1 (**C**) areas presenting Hypoxyprobe-related fluorescence. (**D**) Western blots of HIF-1α from hippocampi extracted from HBO-treated and control 5XFAD mice and wt littermates. (**E**) Quantification of Western blots in (**D**), presented as percentage of wt control, normalized to GAPDH levels (n = 4–5/group). Two-way ANOVA and post-hoc Fisher LSD tests were performed. Values represent means ± SEM. *P < 0.05, ** *P* < 0.01, *** *P* < 0.001.

We next investigated whether the changes in CBF following HBOT reduced hypoxia in the hippocampal area of 5XFAD mice. Fourteen days following the last session of HBO or control treatment, mice were injected with Hypoxyprobe, a label which is only activated and detectable in hypoxic cells, characterized by a partial pressure of oxygen below 10 mm Hg (<1%). As can be seen in Figure 6, the 5XFAD control mice showed increased reactivity of Hypoxyprobe in hippocampal areas CA3 (3.69-fold increase, *P* = 0.0076; Figure 6A, 6B) and CA1 (8.19-fold increase, *P* = 0.0021; Figure 6A, 6C), as compared with the same areas in their wt counterparts. Remarkably, HBOT significantly reduced the reactivity of Hypoxyprobe in the hippocampal formation of 5XFAD mice in both CA3 (3.11-fold decrease, *P* = 0.0145; Figure 6A, 6C) and CA1 (2.48-fold decrease, *P* = 0.0261; Figure 6A, 6C), relative to 5XFAD control mice.

To corroborate our finding and identify the molecular pathway affected by HBOT, levels of the oxygen tension-dependent transcriptional factor hypoxia inducible factor-1 (HIF-1) were tested. Under normoxic conditions, HIF-1α is degraded. However, under hypoxic conditions, HIF1α is stabilized and its levels are increased [57]. HIF1α levels increased in 5XFAD mice, as compared to wt littermates (+55. 17%, *P* = 0.0003; Figure 6D, 6E). HBOT significantly reduced HIF1α levels in 5XFAD mice (-24.12%, *P* = 0.0056; Figure 6D, 6E). Taken together, these experiments indicate a high degree of hypoxia in the hippocampal area of 5XFAD mice that can be significantly reduced by HBOT. Furthermore, these results demonstrate that HBOT reduced the hypoxic state in 5XFAD mice even 14 days after the last HBO session, suggesting that HBOT induced a long-lasting effect by increasing arteriolar lumen volume and elevating blood flow.

### HBOT improves the performance of 5XFAD mice in behavioral tasks

Finally, we explored if the mitigating effects of HBO treatment on AD pathology are associated with an improvement in the performance of 5XFAD mice in behavioral tasks. We found that HBO-treated 5XFAD mice showed improved nest construction abilities, given how they built nests with higher walls and attained higher nest scores (Supplementary Figure 2). Moreover, the treated mice showed improved exploratory behavior, as compared to control 5XFAD mice (Supplementary Figure 2).

**Figure 7:**
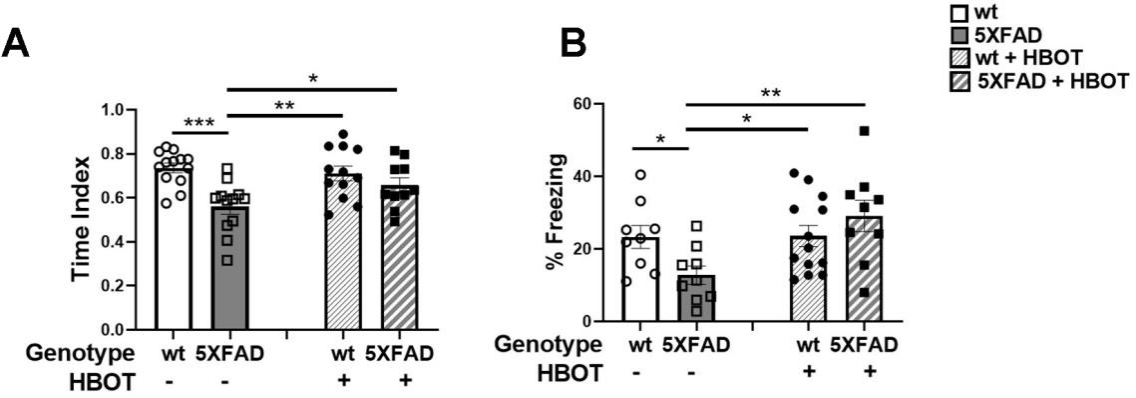
**HBOT improves performance of 5XFAD mice in cognitive tasks.** (**A**) In the Y-maze test, HBO-treated 5XFAD mice showed better spatial memory as reflected in the time index, which is displayed as the ratio (novel /novel + familiar) to time in each arm. (**B**) In the trace fear conditioning assay, mice underwent conditioning involving 6 rounds of tone-shock pairing with a trace interval. On the following day, the mice were exposed to the same context with no exposure to tone or shock. Results of contextual freezing are expressed as the percent of total time spent frozen in the training context. Two-way ANOVA with/without repeated measures and post-hoc Fisher LSD tests were performed. Values represent means ± SEM. *P < 0.05, **P < 0.01, *** *P* < 0.001.

Spatial recognition memory was investigated by testing the natural preference of mice for exploring novel over familiar spatial contexts in a Y-maze test (Figure 7A). Control 5XFAD mice showed a decreased time index in this assay, as compared to their wt littermates (wt control: 0.7367 ± 0.02168 vs. 5XFAD control: 0.5596 ± 0.03382, *P* = 0.00013; Figure 7A), HBOT significantly reversed this trend (5XFAD-HBO: 0.6572 ± 0.03378 vs. 5XFAD-control: 0.5596 ± 0.03382, *P* = 0.0362; Figure 7A). We then tested the effect of HBOT on hippocampus-dependent contextual memory by performing trace fear conditioning [58–60]. Control 5XFAD mice showed impaired contextual memory, relative to their wt littermates, as reflected by lower freezing activity in the training chamber 24 h following conditioning (wt control: 23.29 ± 3.140 % vs. 5XFAD control: 12.81 ± 2.516%, *P* = 0.0377; Figure 7B). Interestingly, this impairment was recovered in HBO-treated 5XFAD mice (5XFAD-HBO: 29.10 ± 4.321% vs. 5XFAD control: 12.81 ± 2.516%, *P* = 0.0019; Figure 7B).

Taken together, these results suggest that HBOT ameliorated the performances of 5XFAD mice in memory and behavioral tasks.

### HBOT increases cerebral blood flow and improves cognitive performances in elderly patients

**Figure 8:**
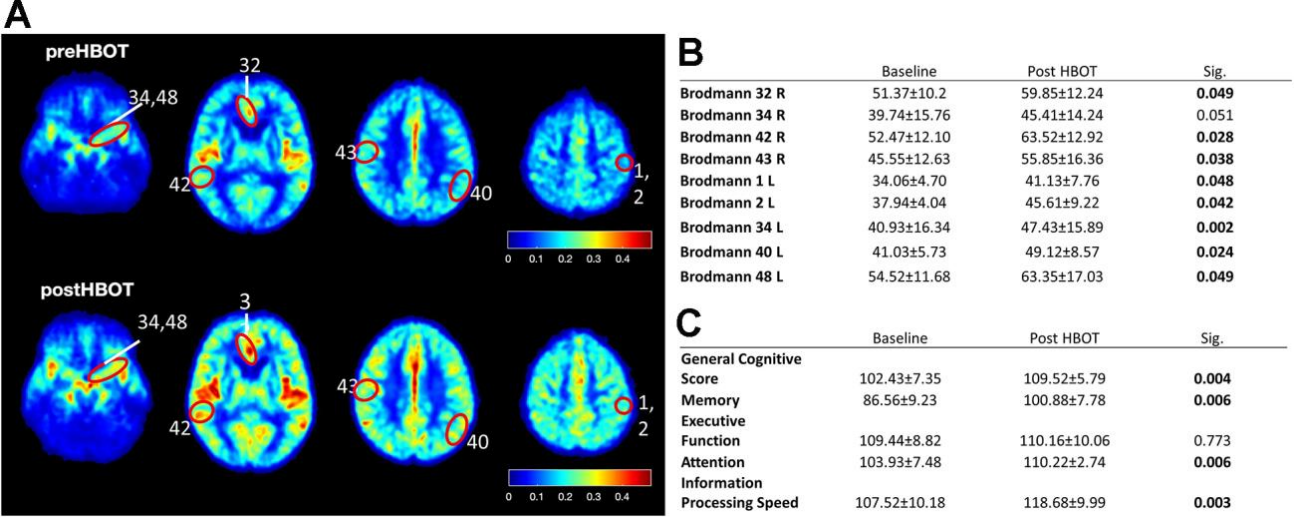
**CBF and cognitive function are improved following HBOT of patients.** CBF and cognitive functions of six patients suffering from memory decline at baseline and following 60 HBOT sessions. (**A**) Average normalized CBF maps (DSC) at baseline and post-HBOT. (**B**) Significant average CBF changes in Brodmann areas at baseline and post-HBOT. (**C**) Average cognitive domain scores (Neurotrax) at baseline and post-HBOT.

To understand whether the ability of HBOT to change CBF and affect cognitive function also applied to elderly people, we performed a human study (NCT02790541) in which six elderly patients (age 70.00 ± 2.68 years) with significant memory loss at baseline (memory domain score < 100) were treated with HBOT (60 daily HBOT sessions within 3 months). CBF and cognitive function were evaluated before and after HBOT. CBF was measured by MRI dynamic susceptibility contrast sequential imaging, while cognitive functions were evaluated using computerized cognitive tests. Following HBOT, there were significant CBF increases in several brain areas, including Brodmann areas 1, 2, 32, 34, 40, 42, 43, and 48 (Figure 8A, 8B). At baseline, patients attained a mean global cognitive score (102.4±7.3) similar to the average score in the general population normalized for age and education level (100), while memory scores were significantly lower (86.6 ± 9.2). Cognitive assessment following HBOT revealed a significant increase in the global cognitive score (102.4 ± 7.3 to 109.5 ± 5.8, p=0.004), where memory, attention and information processing speed domain scores were the most ameliorated (Figure 8C). Moreover, post-HBOT mean memory scores improved to the mean score (100.9 ± 7.8), normalized per age and education level (100). The improvements in these scores correlate with improved short and working memory, and reduced times of calculation and response, as well as increased capacity to choose and concentrate on a relevant stimulus.

Together with our findings using an AD mouse model and the similar effects observed following HBO treatment of stroke and TBI patients, we suggest that HBOT mediates structural changes in blood vessels that increase CBF, reduce brain hypoxia and improve cognitive performance.

## DISCUSSION

In recent decades, the development of drugs for Alzheimer’s disease has primarily targeted beta amyloid and tau pathologies. However, the failures of recent clinical trials suggest that alternative strategies for AD treatment should be considered [61–63]. One promising alternative target is vascular dysfunction, as it is detected in the early stages of AD, correlates with disease progression and affects disease outcome. Improved vascular function and CBF can also improve cognition in the elderly [2, 3, 9, 24, 64]. In this study, we explored this option by manipulating oxygen levels in an Alzheimer’s disease mouse model and in an elderly patient population. The results revealed that increasing oxygen delivery to the brain through HBO treatment improved several aspects of AD pathology, including vascular dysfunction, plaque burden and behavior. HBOT-induced vascular changes also led to increased CBF and reduced cerebral hypoxia that continued weeks after treatment.

This study also demonstrated that HBOT improved vascular parameters in 5XFAD mice. Over the course of one month, the diameters of cortical blood vessels in these mice were reduced by ~8.5% and as a result, the blood flow was decreased by ~20%. This is in accordance with Poiseuille's model, which shows that even minimal changes in vessel diameter can have a dramatic effect on the rate of blood flow (e.g. blood vessel narrowing by 6% reduces blood flow by 22% [65, 66]). In 5XFAD mice, neutrophil adhesion led to stalled capillaries and reduced CBF [15]. Indeed, narrower vessels, as we observed, can accelerate neutrophil adhesion and lead to stalled capillaries. HBOT alleviated the thinning of and increased the flow of blood in cortical blood vessels. Moreover, we showed that the HBOT-induced increase in blood flow was accompanied by an elevation of arteriole luminal diameters and a reduction in hypoxia in 5XFAD mice. Interestingly, reduced hypoxia was detected even 14 days after the last round of HBO treatment, suggesting that structural changes in blood vessels contribute to the reduced CBF of 5XFAD mice and that HBOT can significantly alleviate this reduction, in turn leading to reduced cerebral hypoxia. Hence, HBOT induces changes in the basic pathophysiology of the disease which last at least for 2 weeks after HBOT is completed.

Increased CBF and higher oxygen delivery can improve or boost brain function. HBOT induces cognitive enhancement in both young [67] and elderly healthy subjects through regional changes in CBF [68]. HBOT has also been shown to increase CBF and improve EEG measurements [34], global cognitive scores [33, 69] and PET scans [70] of post-stroke and TBI patients. In the context of Alzheimer’s disease, animal studies have shown that supplementation of oxygen [71, 72] or HBOT [17, 36] results in improved cognitive performance [71] and enhanced cerebral blood volume [72]. Moreover, an immediate increase in CBF due to a reduction in the number of stalled capillaries led to rapid improvement in the performance of 5XFAD mice in spatial and working memory tasks [15]. Recent human studies have shown that HBOT can improve cognitive functions of mild cognitive impairment (MCI), AD and vascular dementia patients [37–39, 73] and ameliorate the reduced brain metabolism of MCI and AD patients [37, 39]. Here, we showed that HBOT increases CBF and improves cognitive function in elderly individuals suffering from significant memory loss, as well as increasing CBF, alleviating cerebral hypoxia and improving behavioral deficits in 5XFAD mice. Yet, as the number of subjects in our human HBOT experiments was relatively small (n=6), expanding these efforts with a larger cohort could strengthen our findings. Taken together, these data suggest that oxygen is a rate-limiting factor for both normal cognitive function and for tissue recovery in Alzheimer’s disease.

Tracking plaques in the somatosensory cortex over time provided insight into the dynamics of plaques *in vivo* and revealed that over one month, the number of plaques in 5XFAD mice nearly doubled, with their volume increasing, suggesting that high and rapid synthesis of plaques occurs in this model. Moreover, small plaques exhibited greater size increments, relative to larger plaques. This is in agreement with other reports [44, 45, 74], although not with the work of Hefendehl et al. [75]. Remarkably, HBOT reduced the appearance of newly-formed plaques and contributed to a reduction in the size of existing medium-sized to large plaques, suggesting that HBOT affects both the synthesis of Aβ and its degradation and clearance. This claim is further supported by our findings that HBOT attenuated the excessive generation of Aβ42 and formation of Aβ plaques by reducing levels of BACE1 (β-secretase) and presenilin1 (a component of the γ-secretase), leading to reduced levels of the C-terminal Aβ fragment (CTFβ, C99) and of the insoluble fraction of Aβ42. In addition, HBOT elevated IDE and LRP1 levels, thus promoting Aβ degradation and clearance. These findings are in accordance with evidence showing that elevating either activity of the degradation pathway by enhancing IDE levels [76, 77] or of clearance pathway by changing LRP1 levels [78] leads to a reduction in amyloid burden. Our finding that the number of microglia per plaque volume increased following HBOT suggests that microglia were recruited to plaques, possibly supporting plaque degradation [79, 80]. Taken together, it seems that HBOT ameliorates the pathways of Aβ42 generation, accumulation and degradation and thus contributes to the reduced appearance of novel plaques and breakdown of existing plaques.

Accumulating evidence has demonstrated that cerebral hypoperfusion and hypoxia result in Aβ generation and accumulation [81–83]. Hypoxia induces Aβ generation by facilitating β- and γ-secretase cleavage of APP [84–87] and Aβ accumulation by decreasing levels of enzymes responsible for Aβ degradation, such as neprilysin (NEP) [88–90] and IDE [91]. Indeed, most AD patients present CAA and degenerative changes affecting arterioles and capillaries, and many show ischemic parenchymal abnormalities. These are the result of structural vascular disease and/or reduction in blood flow in critical brain areas [92, 93]. Eliminating Aβ via the vasculature is an important route for clearing brain Aβ, such that impairment of this process due to cerebral hypoperfusion results in Aβ accumulation and the generation of vascular amyloidosis and dense-core plaques [94]. This positive feedback loop, in which vascular factors increase neurodegenerative deterioration, and vice versa, facilitates disease progression [95]. Here, we have shown that HBOT reduces hypoxia, increases CBF and ameliorates arterioles structure, as well as promoting Aβ degradation and clearance, resulting in reduced amyloid burden. These findings further show the tight interplay between vascular pathology and neurodegeneration and demonstrate the potency of HBOT in countering hypoxia-related neurological conditions, particularly AD.

In summary, we showed here that HBOT offers multi-faceted neuroprotective effects on the complex pathology of Alzheimer’s disease and also improves CBF and cognition in humans. Targeting various pathways involved in the basic pathophysiology of AD may offer a more potent strategy for modifying disease progression. To this point, HBOT has been demonstrated to ameliorate the pathology and improve behavior in three AD mouse models, namely 3xTg-AD, 5XFAD and APP/PS1 mice [17, 36]. Given that HBOT is considered a safe and tolerable treatment currently being used in the clinic, the increasing number of clinical trials showing that HBOT improves cognitive function in patients suffering from chronic brain damage, the pre-clinical studies elucidating mechanisms of HBOT action, and the fact that there is presently no effective intervention for AD, HBOT should be considered as a therapeutic approach to slow the progression or even improve the pathophysiology responsible for this disease.

## MATERIALS AND METHODS

### Mice

Transgenic mice (Tg6799) co-overexpressing FAD mutations of human APP (the Swedish mutation, K670N/M671L; the Florida mutation, I716V; and the London mutation, V717I) and PS1 (M146L/L286V) transgenes under transcriptional control of the neuron-specific mouse Thy-1 promoter [40] were used. Hemizygous transgenic mice were crossed with C57BL/6 breeders for 10 generations. Genotyping was verified by PCR analysis of tail DNA. All animal experiments followed the “Principles of laboratory animal care” (NIH publication No-86-23 revised 1985) and were performed in accordance with animal protocols approved by the Tel Aviv University Animal Care Committee. Reporting was in accordance with ARRIVE guidelines.

### Hyperbaric oxygen therapy (HBOT)

Six month-old male heterozygous 5XFAD mice and wild type (wt) C57BL/6 littermate mice were randomly assigned to two groups: HBO-treated and controls (exposed to normobaric conditions). For HBOT, animals were administered 100% oxygen at a pressure of 2 ATA in a custom-made monochamber (Supplementary Figure 3) intended for small animals for 60 minutes per day, 5 days a week for 4 weeks (i.e., 20 treatments). Before compression was initiated, the monochamber was washed with 100% oxygen for 5 min to enrich oxygen content. Compression and decompression were performed gradually over 5 min. Oxygen levels inside the chamber following compression reached saturation of ≥96%, as measured by an oxygen analyzer (320BRC model, Teledyne Analytical Instruments). Animals in the control group were placed inside the monochamber for 60 min at 1 ATA without additional treatment (n=20).

### Behavioral testing

The effects of HBOT on mouse memory and behavior were evaluated using a battery of behavioral tests. The nest building test was administered before and after 1 month of HBOT or control treatments. Tests were performed 24 h following the last HBOT/control treatment and was ended 48 h prior to sacrifice, to reduce stress.

### Y-maze test

Mice were placed at the distal end of the entrance arm and allowed to explore the maze for 5 min with only the familiar arm available for exploration. After a 2 min delay, the mice were reintroduced into the maze with two arms (familiar and novel) available for exploration and documented for 2 min. The ratios of time spent and the frequency of visits to the novel arm were calculated as the time or visit frequency in the novel arm divided by the sum of time or visit frequency in both the novel and familiar arms. The maze was cleaned with 40% ethanol between sessions. Arms were changed randomly between animals, yet were kept similar for each animal.

### Trace fear conditioning

Mice were placed in a training chamber. After a 120 s baseline period, the mice received five pairings of the Conditional stimulus (CS: tone, 5 kHz, 70 dB) and Unconditional stimulus (US; shock 2 s, 0.7 mA). The CS and US were separated by a 18 s empty trace interval, which increased hippocampal dependency. The inter-trial interval was set at 90 s [96]. The training chamber was wiped with 40% ethanol between sessions. Twenty-four hours later, the mice were again placed in the training chamber and the percentage of behavioral freezing (i.e., the absence of all but respiratory movement) during a 5 min test session was measured (contextual memory) using a FreezeFrame automated scoring system (Coulbourn Instruments).

### Biochemical and histological analyses

Mice were anesthetized with ketamine and xylazine and perfused transcardially with PBS. Brains were then excised and halved and each hemisphere was further processed for either biochemical or histological analysis, as outlined below. Antibodies used in this project are listed in Supplementary Table 1.

### Immunochemistry

One brain hemisphere was fixed overnight with 4% paraformaldehyde in 0.1 M phosphate buffer (pH 7.4) and then placed in 30% sucrose for 48 h. Frozen coronal sections (30 μm) were then cut on a sliding microtome, collected serially and stored in cryoprotectant (containing glycerine, ethylene glycol, and 0.1 M sodium phosphate buffer, pH 7.4) at -20° C until use. Three-four free-floating sections per animal at bregma −1.35 mm and bregma −2.78 were immunostained with the following primary antibodies: biotinylated mouse anti-Aβ 17-24 (4G8, 1:200; Signet Laboratories), and FITC-conjugated mouse anti-smooth muscle actin (α-SMA, 1:1000; Sigma-Aldrich). Sections were first blocked with 10-20% normal goat serum in PBST (0.1% triton-x-100 in PBS) for 2 h at room temperature, and then incubated for 24 h at 4° C with the primary antibodies (dissolved in 2% (w/v) normal goat serum in PBST). Binding of the primary antibodies was visualized by incubating the sections for 1.5 h at room temperature with secondary antibodies, depending on the primary antibodies used. The sections were then mounted on slides coated with dry gelatin. Aβ staining was similarly performed, except that prior to blocking, the sections were incubated with 70% formic acid for 6 min to increase antigen retrieval before antibody staining.

The sections were visualized using an EVOS FL microscope (Thermo Fisher; 4× and 20x magnifications) or a confocal scanning laser microscope (SP8, LEICA). Control experiments revealed no staining in sections that lacked the first antibodies and were used to determine the threshold for intensity quantification. Intensity of the immunofluorescent staining above threshold level was calculated with the Image-Pro Plus system (version 5.1, Media Cybernetics).

For SMA analysis, images were acquired from arterioles in cortical and hippocampal areas using a confocal microscope (Leica, SP8, 40× magnification). All images were acquired in a random manner blinded to subject. Vessels showing positive SMA signals and ranging in size from 10-50 μm were analyzed with FIJI ImageJ software (National Institutes of Health, Bethesda, MD). A minimum of 10 and 25 arterioles were imaged for cortical and hippocampal areas per animal, respectively. For hippocampal arterioles, luminal diameter was measured as an average of the inner diameters across the section. For penetrating arterioles, diameter was assessed by determining the minimum axis of the ellipse, which is the arteriolar minor axis. Wall thickness of the medial layer was determined by measuring the external and luminal diameters and then taking half of the difference.

### Hypoxyprobe staining

On the day the mice were sacrificed, half of each treatment group were injected intraperitoneally (i.p.) with 60 mg/kg of Hypoxyprobe 1 (pimonidazole hydrochloride, Hypoxyprobe, Burlington, MA) 30 min before tissue harvesting to detect hypoxia. Pimonidazole is distributed to all tissues, including the brain, but only forms stable adducts with thiol groups in proteins, peptides and amino acids found in hypoxic cells under conditions of partial pressure of oxygen below 10 mm Hg at 37° C. Brains were excised and one brain hemisphere was fixed in paraformaldehyde followed by 30% sucrose, as described above. Free-floating sections were immunostained with rabbit anti-pimonidazole antibodies (1:500) and visualized by Alexa Fluor 488-conjugated goat anti-rabbit secondary antibodies (1:1000). The sections were then mounted on dry-gelatin-coated slides and visualized using an EVOS FL microscope (Thermo Fisher; 4× magnification).

### Immunoblotting

One brain hemisphere was snap-frozen in liquid nitrogen and stored at -80° C until use. The hippocampus was excised on ice at 4° C. Proteins were dissolved in 200 μl lysis buffer containing 7.5 mM HEPES, pH 7, 1.5 mM EDTA, 1.5 mM EGTA, 0.375 mM DTT, protease inhibitor cocktail (P8340, Sigma), phosphatase inhibitor cocktail (P5726, Sigma) and 2.5% SDS (Amresco Pure, Technology Grade). Protein concentrations were determined using Bradford reagent (Bio-Rad Laboratories, Hercules, CA). Equal amounts of protein were separated on 4–20% Bis-Tris gels (BioRad) and transferred to nitrocellulose membranes. Membranes were blocked overnight in 5% (w/v) non-fat milk in 0.1% Tween 20 in Tris-buffered saline (TBS). After blocking, the membranes were incubated for 1 h at room temperature with primary antibodies specified in Supplementary Table 1.

The membranes were then washed in Tween–TBS for 20 min and incubated at room temperature with specific horseradish peroxidase-conjugated secondary antibodies as specified in Supplementary Table 1 for 60 min. Antibody binding was revealed using enhanced chemiluminescent substrate (Pierce) and band intensity was quantified with ImageQuant TL software (Amersham). Glyceraldehyde 3-phosphate dehydrogenase (GAPDH) levels were used to verify uniform loading of the samples.

### Aβ enzyme-linked immunosorbent assay (ELISA)

Protein extraction was prepared by sequential ultracentrifugation of brain sub-region homogenates. Frozen tissues of hippocampus from 5XFAD mice were weighed and mechanically homogenized in four volumes of ice-cold TBS supplemented with protease inhibitors (Sigma, P8340), phosphatase inhibitors (Sigma, P5726) and 2 mM EDTA. Samples were ultracentrifuged at 350,000 g for 30 min at 4° C, and the collected supernatant was labeled as the protein TBS fraction (TBS). The pellet was dissolved in 200 μl of 70% formic acid supplemented with phosphatase and protease inhibitor cocktails and rotated for 2 h at RT. The tubes were then centrifuged at 350,000 g for 30 min at 4° C. The supernatant was collected and 20 volumes of 1M Tris were added to generate protein FA fraction (FA). Levels of Aβ42 and Aβ40 in the hippocampus were quantified with a β-amyloid x-42 ELISA Kit (Biolegend, 842401) and a β-amyloid x-40 ELISA Kit (Biolegend, 842301), respectively. Levels of Aβ42 and Aβ40 are presented as pg/ml Aβ to total protein mg/ml protein (pg/mg).

### Cranial window generation and two-photon imaging

Cranial windows were generated as previously reported [97]. Carprofen (Pfizer, 15 μg/25 g mouse) analgesia was administered sub-cutaneously prior to surgery. Mice were anesthetized with isoflurane (5% for induction, 1–2% thereafter), the scalp and connective tissues were excised, and the skull was covered with cyanoacrylamide. A 3-mm diameter craniotomy was performed over the barrel cortex (primary somatosensory cortex; Bregma: rostral −1.5, lateral 3 mm) and a custom-made 3 mm cover glass was placed and sealed with cyanoacrylate glue. The dry glue was covered with dental acrylic. An aluminum metal bar with two traded holes was attached to the skull. During surgery and until full recovery, the mouse was kept at 37° C using a heated plate. Ringer’s solution (1 ml) was administered sub-cutaneous after surgery.

For imaging, mice were anesthetized with isoflurane (5% for induction, 1.5% thereafter) in pure oxygen. The mice were mounted in a custom-made stage using a pre-attached head bar, and their temperature was maintained at 37° C using a heated plate. Imaging was conducted with a custom-modified two-photon laser-scanning microscope based on a Sutter MOM apparatus controlled by MPScope 2.0 software [98] using 810 nm excitation.

For plaque imaging, mice (n = 3-4 per group) were injected i.p. with 10 mg/kg methoxy-X04 (TOCRIS, 4920, batch no: 2A/175057; 5 mg/ml in 10% DMSO, 45% propylene glycol, 45% PBS, pH 7.5) 24 h prior to each imaging session [99]. The same 3-4 fields of view, 200-400 μm in depth, were imaged per animal before and after 1 month of exposure to HBOT or control treatments and plaques were sorted according to their volume. 3D reconstruction of the Methoxy-X04 plaques was done using Imaris V7.1.1 software (Imaris, RRID:SCR_007370).

For visualization of blood plasma, mice were injected with 20 μl FITC (5% w/v, i.v.; Sigma-Aldrich) via the infraorbital vein before imaging. Blood cell velocity was measured in blood vessels using arbitrary scan patterns, as previously described [100]. The same vessels were assessed prior to and following 1 month of HBO/control treatment. Procedures for blood flow measurement and analysis have been described previously [54]. The diameter of a blood vessel and the velocity of RBCs therein can be combined to determine absolute RBC flux, which provides a complete description of blood flow in each vessel [54, 101] and is given by:

$$Flow=\;\frac{\pi }{8}VD^{2}$$

where V is the time-averaged RBC velocity at the center line of the vessel, and *D* is the lumen diameter [54].

### Human subjects

The study population comprised adults (5 males, 1 female) with significant memory decline aged 64 years and older, who lived independently and who were in good functional and cognitive status. The study was performed between 2016-2020 at the Shamir (Assaf-Harofeh) Medical Center, Israel. Patients included in the study did not have cardiac or cerebrovascular ischemia histories for the last year prior to inclusion. Exclusion criteria included previous treatment with HBOT for any reason during the last three months, any history of malignancy during the last year, any pathological cognitive decline, severe chronic renal failure (GFR < 30), uncontrolled diabetes mellitus (HbA1C > 8, fasting glucose > 200), taking immunosuppressants, MRI contra-indications, active smoking or pulmonary diseases. Recruitment was based on social media posts and advertisements. The study protocol was approved by the Institutional Review Board of Shamir Medical Center (0172-15-ASF). Clinical trial registration: NCT02790541.

### HBO treatment of human subjects

HBOT protocol was administered in a multiplace Starmed-2700 chamber (Haux, Germany). The protocol consisted of 60 daily sessions at 5 sessions per week within a three-month period. Each session included breathing 100% oxygen by mask at 2ATA for 90 min with 5 min air breaks every 20 min. Compression and decompression rates were 1 m/min.

### Cognitive assessments

Cognitive functions were assessed using the NeuroTrax computerized testing battery, supervised by a certified neuropsychologist. NeuroTrax tests evaluate multiple aspects of brain cognitive function, including memory, executive function, attention, information processing speed, visual spatial, verbal and motor skills. The global cognitive domain evaluates the overall performance in all of the above categories. Cognitive domain scores were normalized for age, gender and education.

Participants completed validated alternate test forms of the NeuroTrax test battery at baseline and post-HBOT to allow for iterative administration with minimal learning effects. Test-retest reliability of the tests was found to be high in both normal and injured populations without significant learning effects, except in the verbal fluency (VF) and visual spatial (VS) domains that were not evaluated in the current study.

### MRI scans

MRI scans were performed on a MAGNETOM Skyra 3T scanner, configured with 20 channel receiver head coils (Siemens Healthcare, Erlangen, Germany). Fifty T2*-weighted gradient-echo echo planar imaging (EPI) volumes were acquired. Two repetitions were performed before a bolus injection of gadolinium-DTPA (Gd-DTPA, 0.2 ml/kg, administered at 5 ml/sec), and 48 repetitions were performed after injection of Gd-DTPA. Sequence parameters were TR: 2,300 ms; TE: 40 ms; flip angle: 30°; voxel size: 1.8 x1.8, matrix: 128 x 128; number of slices: 25; and slice thickness: 3.9 mm. Pre-processing of the perfusion MRI data was preformed using SPM software (version 12, UCL, London, UK) and included motion correction, and co-registration with MPRAGE T1 images. Individual gray matter (GM) and white matter (WM) segmentation of T1 anatomy was also performed to extract mean perfusion values. Whole-brain quantitative perfusion analysis was done as described [102, 103]. CBF values were normalized.

### Statistical analysis

Statistical analyses performed are described in each figure legend. Data from nest building test and two-photon microscopy experiments were analysed by two-way ANOVA repeated measures, followed by a Fisher LSD *post hoc* test. Comparing analysis of all 4 treatment/genotype groups was done using two-way ANOVA followed by a Fisher LSD *post hoc* test. Student’s *t*-test and t-test with Welch’s correction were used to compare two groups of data. Data are presented as mean ± standard error of the mean (SEM). For all analyses, statistical significance was accepted at *P <* 0.05 and trends were defined when *P <* 0.10. For MRI analysis and cognitive tests of humans, the Kolmogorov-Smirnov test, paired T-tests, and FDR corrections were used. For MRI analysis, spatial normalization to the MNI (Montreal Neurological Institute) atlas, followed by gaussian smoothing of 4 mm FWHM (full width at half maximum) was applied.

## Supplementary Material

Supplementary Methods

Supplementary Figures

Supplementary Table 1
