# Supplementary material for: Hyperbaric oxygen therapy alleviates vascular dysfunction and amyloid burden in an Alzheimer’s disease mouse model and in elderly patients
Source: Aging (Albany NY). 2021 Sep 9;13(17):20935–61. doi: 10.18632/aging.203485 (PMC8457592; doi:10.18632/aging.203485)
Supplement: Supplementary Methods [file aging-13-203485-s001.pdf]

SUPPLEMENTARY METHODS

Quantifying plaque-associated microglia

For analysis of plaque-associated microglia, co-staining of Iba1 and amyloid plaques was conducted using biotinylated mouse anti-Aβ 17-24 (4G8, 1:200; Signet Laboratories) and rabbit anti-Iba1 antibodies (1:700, Wako) (as detailed in 'Immunochemistry' section) and the number of microglia associated to plaque and the volumes of plaque (μm^3) in the CA1 area were measured with FIJI Image J software (National Institutes of Health, Bethesda, MD). Then, microglia number was normalized per plaque volume.

Behavioral testing

The effects of HBOT on memory and behavior in mice were evaluated using a battery of behavioral tests. Tests were performed 24hr following the last HBO / control treatment and finished 48 h prior to sacrifice to reduce stress.

Nest building test

The ability of 5XFAD and wt mice to build a nest over night was assessed prior to and following 1 month of HBOT or control normobaric conditions. Mice were placed individually into cages with bedding covering the floor to a depth of 0.5 cm and 2 round pressed cotton batting ('nestlets') about one hour before the dark phase, and the results were assessed the next morning. Results were assessed by scoring nest construction according to the established system of Deacon [1, 2] with a 5-point scale and measuring nests height.

Open field test

Animals were placed in the center of an open field (40 cm × 40 cm × 30 cm) and exploration was assessed for 5 min. Cages were cleaned with ethanol following each session.

Antibodies list

All antibodies used are detailed below in Supplementary Table 1.

REFERENCES

1. Deacon R. Assessing burrowing, nest construction, and hoarding in mice. J Vis Exp. 2012; e2607. https://doi.org/10.3791/2607 PMID:22258546

2. Deacon RM. Assessing nest building in mice. Nat Protoc. 2006; 1:1117–19. https://doi.org/10.1038/nprot.2006.170 PMID:17406392
