# Supplementary material for: Hyperbaric oxygen therapy alleviates vascular dysfunction and amyloid burden in an Alzheimer’s disease mouse model and in elderly patients
Source: Aging (Albany NY). 2021 Sep 9;13(17):20935–61. doi: 10.18632/aging.203485 (PMC8457592; doi:10.18632/aging.203485)
Supplement: Supplementary Figures [file aging-13-203485-s002.pdf]

SUPPLEMENTARY FIGURES

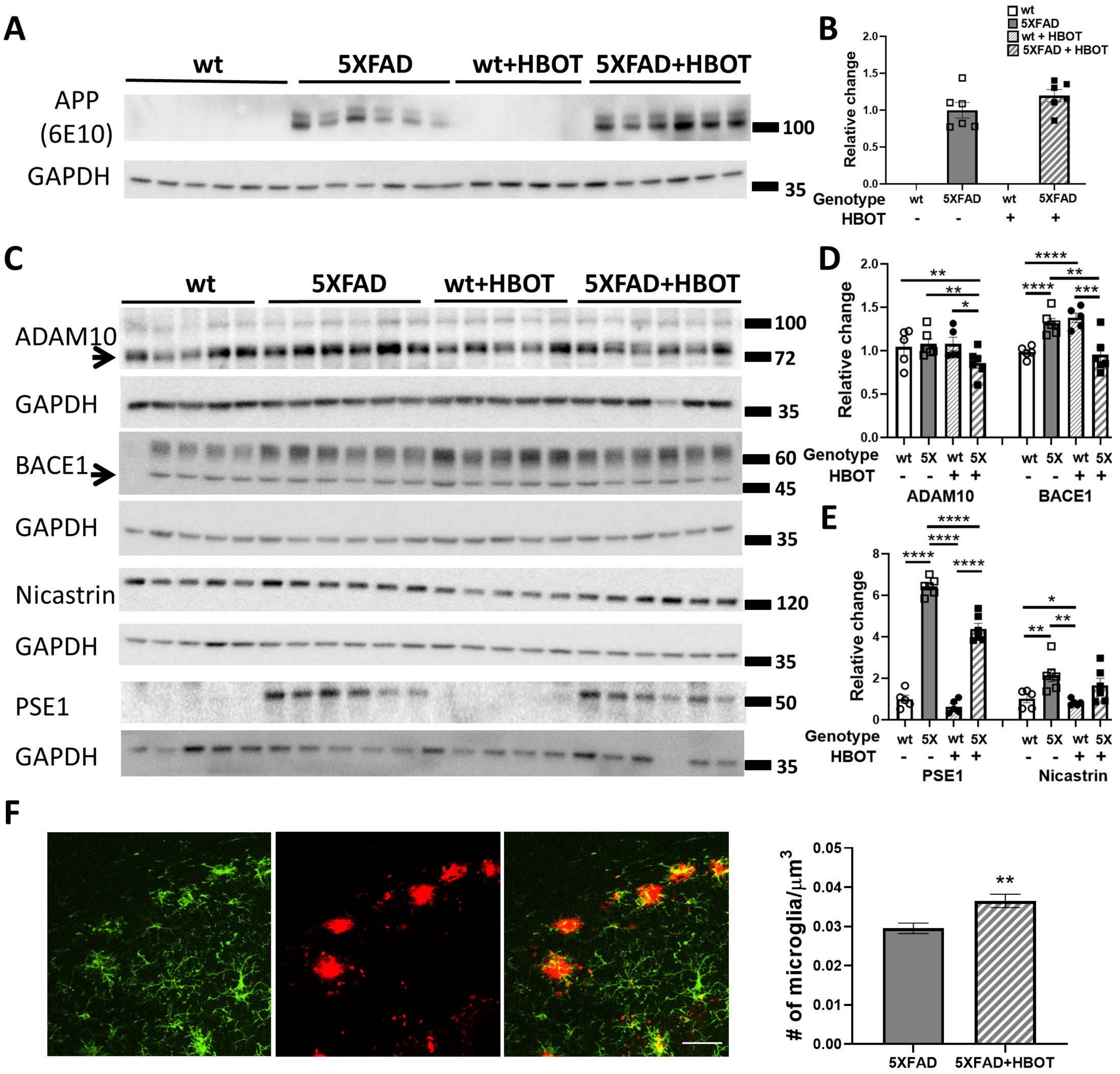

Supplementary Figure 1. Western blots of proteins involved in the APP processing pathway in hippocampi extracted from HBO-treated and control 5XFAD mice and wt littermates. (**A**) Representative immunoblots of the full APP protein detected by 6E10 antibodies. (**B**) Quantification of western blots in (**A**), presented as percentages of levels in 5XFAD controls, normalized to GAPDH levels (n = 4–5/group). (**C**) Representative immunoblots of APP-processing enzymes (ADAM10 and BACE1) and proteins comprising the γ-secretase complex (PSE1 and nicastrin). (**D**, **E**) Quantification of the western blots in (**C**), presented as percentages of levels in wt controls, normalized to GAPDH levels (ADAM10: -37.67%, P = 0.0082; BACE1: -18.16%, P = 0.0011; PSE: -25.62%, P < 0.000001; n = 4–5/group). Two-way ANOVA and post-hoc Fisher LSD tests were performed. (**F**). Values represent means ± SEM. * $P < 0.05$, ** $P<0.01$, *** $P < 0.001$, **** $P < 0.0001$. On the left panel, representative images of microglia (Iba-1 staining, green) and plaques (anti-Aβ 4G8 staining, red) in the hippocampal CA1 of a 5XFAD mouse. Scale bar 40 µm. On the right panel, quantification of the number of plaque-associated microglia normalized to plaque size (µm^3) in control (N= 1108 plaques) and HBOT 5XFAD (N=907 plaques) mice (n=5 per group).

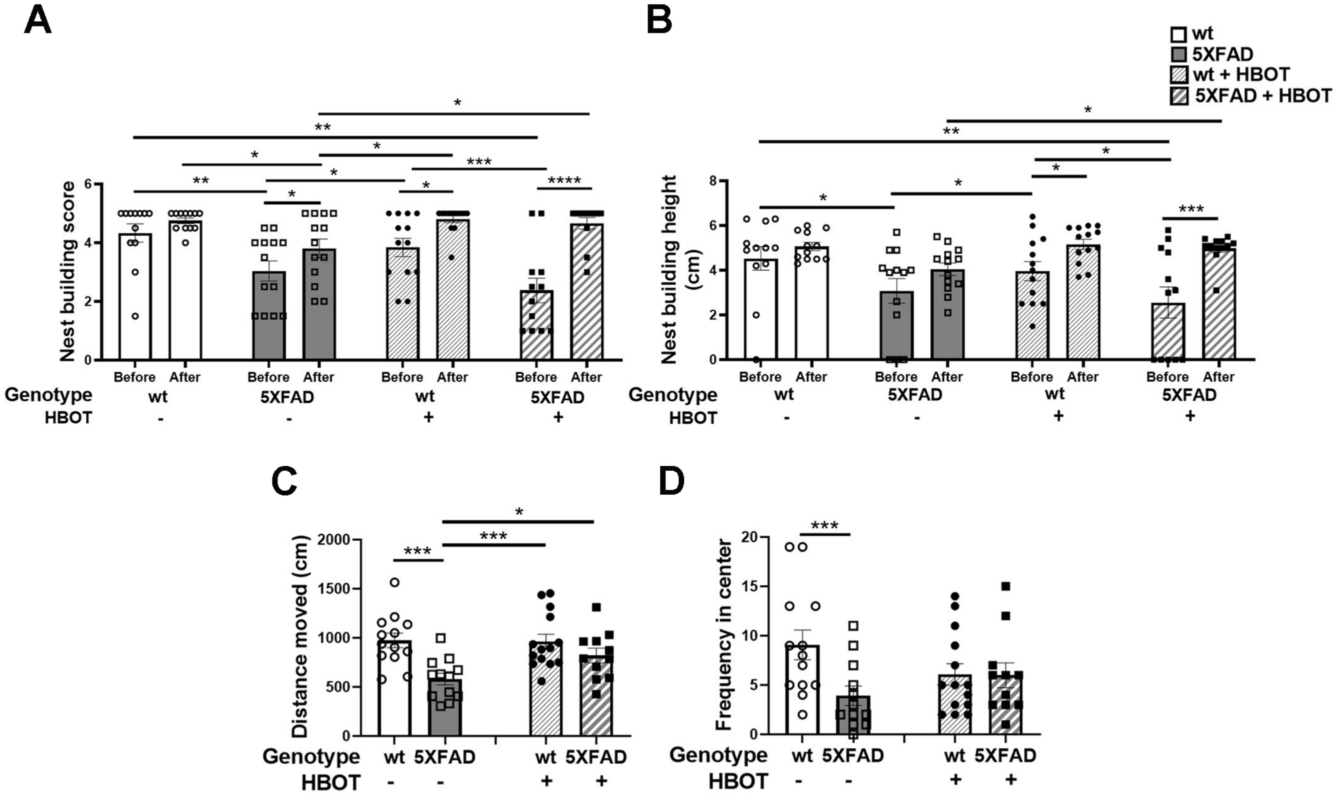

Supplementary Figure 2. HBOT improves performance of 5XFAD in behavioral tasks. Overnight nest building was assessed by nest building score (**A**) and height (**B**) before and after a month of HBO or control normobaric treatment of 5XFAD mice and their wt littermates. (**C**, **D**) In the open field test, HBO-treated 5XFAD mice show higher locomotive activity than control 5XFAD mice, as demonstrated by distances covered (**C**). No changes were observed between HBO-treated 5XFAD mice and control mice in terms of navigation in the open field, as reflected by the number of times the center of the arena was crossed (**D**). Values represent means ± SEM. * $P < 0.05$, ** $P<0.01$, *** $P < 0.001$, **** $P < 0.0001$.

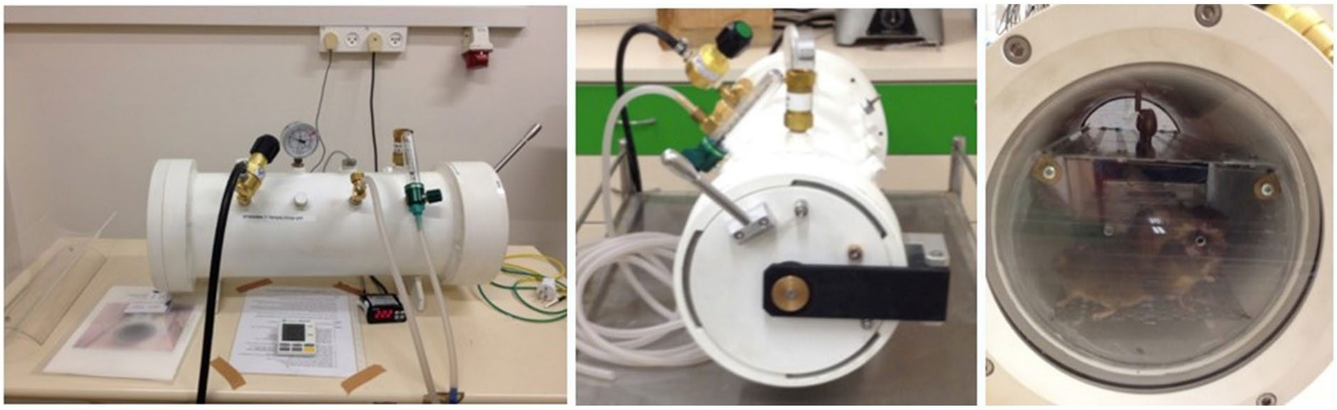

Supplementary Figure 3. Hyperbaric oxygen therapy chamber for small animals. A custom-made chamber for small animals was used to expose 5XFAD mice to HBOT. Oxygen levels in the chamber reached saturation of ≥96%, as measured by an oxygen analyzer (model 320BRC, Teledyne Analytical Instruments). Left: front view; middle: side view; right: side view with open door.
