# Supplementary material for: Hyperbaric oxygen therapy alleviates vascular dysfunction and amyloid burden in an Alzheimer’s disease mouse model and in elderly patients
Source: Aging (Albany NY). 2021 Sep 9;13(17):20935–61. doi: 10.18632/aging.203485 (PMC8457592; doi:10.18632/aging.203485)
Supplement: Supplementary Table 1 [file aging-13-203485-s003.pdf]

Supplementary Table

Supplementary Table 1. List of antibodies used for this research.

Antibody	Application	Source	Dilution
**Biotin-conjugated anti-Aβ mouse mAb (4G8)**	IHC	Covance, SIG-39240	1:200
**FITC conjugated mouse anti- smooth muscle actin (α-SMA**	IHC	Sigma-Aldrich, F3777	1:1,000
**Streptavidin-conjugated goat anti-rabbit Ab**	IHC	Invitrogen, S11226	1:1,000
**488-conjugated goat anti- rabbit**	IHC	Jackson ImmunoResearch Laboratories	1:1,000
**Rabbit anti- Iba1**	IHC	WAKO, 019-19741	1:700
**HRP- conjugated Goat anti-rabbit Ab**	WB	Jackson ImmunoResearch Laboratories, 111-035-144	1:10,000
**HRP- conjugated Goat anti-mouse Ab**	WB	Jackson ImmunoResearch Laboratories, 115-035-003	1:15,000
**Mouse anti-GAPDH**	WB	Abcam, ab9484	1:1,000
**Rabbit anti-ADAM10 pAb**	WB	Abcam, ab1997	1:500
**Rabbit anti-BACE1**	WB	Sigma-Aldrich, B0681	1:1,000
**Rabbit anti-Nicastrin mAb**	WB	Cell Signaling D65G7 #9447	1:5,000
**Mouse anti-Presenilin 1 mAb**	WB	Chemicon, Ab 5232	1:500
**Rabbit anti Insulin degrading enzyme**	WB	Abcam, ab32216	1:1,000
**Rabbit anti- Low density lipoprotein receptor- related protein 1**	WB	Abcam, ab92544	1:1,000
